# A qualitative analysis of a consensus process to develop quality indicators of injury care

**DOI:** 10.1186/1748-5908-8-45

**Published:** 2013-04-18

**Authors:** Niklas Bobrovitz, Julia S Parrilla, Maria Santana, Sharon E Straus, Henry T Stelfox

**Affiliations:** 1 Department of Community Health Sciences, University of Calgary, Calgary, Canada; 2 Department of Medicine, Institute of Public Health, W21C Research and Innovation Center, University of Calgary, Calgary, Canada; 3 Department of Critical Care Medicine, University of Calgary, Calgary, Canada; 4 Department of Medicine, Saint Michael’s Hospital, University of Toronto, Toronto, Canada; 5 Departments of Medicine and Community Health Sciences, Institute for Public Health, University of Calgary, 3280 Hospital Drive NW, Calgary, AB T2N 4Z6, Canada

**Keywords:** Quality indicator, Wounds and injuries, Delphi technique, Quality improvement, RAND/UCLA Appropriateness method

## Abstract

**Background:**

Consensus methodologies are often used to create evidence-based measures of healthcare quality because they incorporate both available evidence and expert opinion to fill gaps in the knowledge base. However, there are limited studies of the key domains that are considered during panel discussion when developing quality indicators.

**Methods:**

We performed a qualitative content analysis of the discussions from a two-day international workshop of injury control and quality-of-care experts (19 panel members) convened to create a standardized set of quality indicators for injury care. The workshop utilized a modified RAND/UCLA Appropriateness method. Workshop proceedings were recorded and transcribed verbatim. We used constant comparative analysis to analyze the transcripts of the workshop to identify key themes.

**Results:**

We identified four themes in the selection, development, and implementation of standardized quality indicators: specifying a clear purpose and goal(s) for the indicators to ensure relevant data elements were included, and that indicators could be used for system-wide benchmarking and improving patient outcomes; incorporating evidence, expertise, and patient perspectives to identify important clinical problems and potential measurement challenges; considering context and variations between centers in the health system that could influence either the relevance or application of an indicator; and contemplating data collection and management issues, including availability of existing data sources, quality of data, timeliness of data abstraction, and the potential role for primary data collection.

**Conclusion:**

Our study provides a description of the key themes of discussion among a panel of clinical, managerial, and data experts developing quality indicators. Consideration of these themes could help shape deliberation of future panels convened to develop quality indicators.

## Introduction

Improving the quality of healthcare is a widely recognized international challenge [1-3]. However, to improve care, the quality of care must first be measured using valid and reliable tools. In response to this need, efforts to develop and implement evidence-based measures of quality have increased [4,5].

Quality indicators are one type of quality measure that have been advocated by professional health-provider organizations*,* institutions, accrediting bodies, and government agencies, e.g., Agency for Healthcare Research and Quality, (AHRQ) [6,7]*.* These measures compare actual patient care to ideal criteria and can be used by providers, policy makers, and researchers to identify problem areas, tailor interventions, and track subsequent improvements. Thousands of indicators have been proposed in the literature and are used in practice [8]. However, very few indicators are evidence-based and have been broadly implemented with routine reporting and analysis for specific medical problems or healthcare services [8-10].

Frameworks for developing and assessing quality indicators have been published [11,12]. A 2012 review described 10 methodological approaches to the guideline-based development of quality indicators, but concluded that it is unclear which method produces the ‘best’ quality indicators [12]. The majority of proposed frameworks suggest multi-step processes to indicator development and evaluation including the utilization of consensus methodologies to incorporate both the best available evidence and expert opinion to fill gaps in the knowledge base [11,12]. However, there is limited research describing the key themes and points of discussion among consensus panels developing quality indicators.

Therefore, we analyzed transcripts from a consensus process to develop quality indicators of injury care [13] to identify the factors affecting the development, selection, and refinement of indicators. Our analysis was designed to further inform existing frameworks for creating standardized, evidence-based measures of health system performance.

## Methods

The current study is a qualitative content analysis of the discussions from a two-day international workshop of injury control and quality of care experts held April 2011 in Calgary, Canada. A verbatim transcript of the workshop discussion was analyzed by two investigators (NB, JSP).

The workshop was modeled on the RAND/UCLA Appropriateness Method with the goals of; developing indicators to measure the quality of injury care; prioritizing the indicators; agreeing on an indicator refinement process; establishing an indicator evaluation process; and generating a knowledge translation strategy.

Quality indicators were gathered from two sources. First, through a systematic review of the literature that identified published quality indicators for evaluating adult trauma care and summarized the evidence about their reliability, validity, and implementation [8]. Second, from an international audit of trauma center quality improvement practices that reported quality indicators used in clinical practice [14].

A list of potential quality indicators was compiled from the two sources, duplicates deleted, and presented to a 19 member multi-disciplinary expert panel. We used a purposive sampling strategy to ensure diverse expert (emergency medical services, sub-specialty hospital care, rehabilitation, secondary injury prevention, measuring healthcare quality, organizational leadership) and geographic (Canada, United States, Australia, including urban and rural) representation of key perspectives in injury control. Panelists were nominated by members of the American College of Surgeons Trauma Quality Improvement Program, American College of Surgeons Advanced Trauma Life Support Committee, and the Trauma Association of Canada. Nominated panelists were sent letters of invitation with details of the time requirements and program details and were asked to suggest other experts in related disciplines. Of the 23 nominees offered membership to the panel, 19 (83%) agreed to participate.

Panelists used an electronic survey tool to independently score the indicators over two rounds of review. All the indicators were then evaluated for final selection at the workshop through deliberation and agreement on detailed specifications for the indicators including their definitions, numerators, denominators, and risk adjustment strategies.

Given the diversity of expertise and the multi-national composition of the panel, it offered an excellent opportunity for a case study of the multi-disciplinary consensus process often utilized [12] to develop quality indicators for healthcare. Therefore, we chose to qualitatively analyze this panel’s workshop discussion. The workshop proceedings were recorded (10.5 hours), transcribed verbatim, and analyzed inductively by two of the authors (NB, JSP) using the methods of constant comparative analysis. A process of thematic, open, axial, and selective coding was used to extract themes regardless of pertinence to a physical linguistic unit; word, phrase, sentence, or paragraph [15]. Categories or themes based on valid inference and interpretation were produced out of grouped codes [15]. A coding framework was created to ensure inter-coder reliability and coding consistency. Memos were used to provide evidence of the analytic process and the decisions made to develop concepts and compared by the two researchers. This iterative method was used to ensure consistency, inter-coder agreement, trustworthiness, and to ascertain themes. This was done exhaustively until saturation was obtained and no new themes surfaced. Reliability and consistency was checked regularly through iterative and constant comparison approaches to the data set (transcript). Validity was ensured through the creation of a coding manual to ensure inter-coder reliability and coding consistency. Frequency counts—medians with interquartile ranges (IQR)—were calculated for the number of times each panelist spoke during workshop discussion.

Ethics approval was obtained from the Conjoint Health Research Ethic Board. Participants provided written consent to have the workshop proceedings recorded and transcribed for Content Analysis. This manuscript complies with the RATS guidelines for reporting qualitative research [16].

## Results

During the two-day workshop, panelists spoke a median of 71.5 times (interquartile range = 49, 132) (Table 1). The four major themes that emerged during the workshop discussion to develop a standardized set of injury care quality indicators included: clear purpose and goals; incorporating evidence, expertise, and patient perspectives; contextual considerations and variations between centers; and data collection and management. The coding framework (Additional file 1), detailed description of the codes, categories, and themes (Additional file 2) and select quotes are provided as supporting documents.

**Table 1:** Panelist expertise and speaking frequency^*^

**Expertise classification**	Number of panelists^**^	**Speaker count**
• Emergency medical systems	5	780
• Organizational leadership	6	833
• Subspecialty hospital care	5	630
• Rehabilitation and secondary injury prevention	2	134
• Measuring healthcare quality	5	722

^*^The workshop was facilitated by two physicians with expertise in health policy and management and knowledge translation.

^**^Number of participants adds up to more than 19 as a result of some panelist’s expertise in more than one area.

### Clear purpose and goals of the quality indicators

The panelists agreed from the outset that the ultimate goal of using indicators was to improve patient outcomes:

‘…I would submit that the end that we seek here is to try and draw the line between the indicator in question and its measurability and whether or not the patients are going to have better outcomes as a consequence.’

This primary goal was reiterated throughout the workshop and served as the fundamental criterion for indicator selection. Although barriers to indicator development and implementation were identified (e.g., availability of existing data), panelists focused on selecting ‘ideal’ indicators, designed to address important problems in injury care, regardless of the current data availability.

Panelists had the common understanding that the indicators would serve to flag instances when care was suboptimal to promote improvement in those service areas. As a result, pairing indicators that measure structural aspects of care with ones measuring processes of care was repeatedly raised by panelists. This idea was supported by Donabedian’s framework [17] that patient outcomes are not only dependent on the structural aspects of care (e.g., appropriate care protocol), but also on the resulting processes of care (e.g., how well is the care protocol adhered to): ‘[There] needed to be, many times, structural process combos where not only is it useful to have…a mass transfusion protocol in place, but … is it activated and used?’

Participants identified the goal of system-wide improvement. As a result, much of the discussion was geared toward achieving broad implementation and comparability of results to allow for benchmarking*:* ‘…a good performance indicator…should be able to benchmark and say …‘how come hospital X is doing 60% and the other one’s doing 75%?’ Reporting results to drive local quality improvement was also identified as a potential outcome of the indicators:

‘… At one time we had a spike in our pre-hospital cricothyroidotomy rates. We engaged in an extensive discussion with EMS [Emergency Medical Service] providers to scope out the problem, review the cases and come up with a consensus on applying that intervention. That’s an example of where this kind of [local] reporting [was] useful for us.’

A common understanding of each indicator’s focus was vital to selection and development. Once panelists agreed on an indicators focus, they were able to develop explicit definitions and determine the data elements required:

‘…If you’re measuring…the effectiveness of the trauma team to get to the [CT] scanner, we should restrict it to severe traumatic brain injury, and put an hour gap. If we’re measuring access to CT…then you apply the CT head rule, local guidelines, and patients received in CT…within four hours….’

### Incorporating evidence, expertise and patient perspectives

Panelists referred to scientific evidence and their expertise, both clinical and epidemiological, to comment on the magnitude of clinical problems in practice and therefore the relative importance of the indicators addressing that area:

‘…There’s good evidence that appropriate acute pain management decreases physiologic complications, the susceptive stimulation later on, and the patients appreciate it. I think it should be airway, analgesia, breathing, circulation, as opposed to the ABC’s that we normally allude to. I think it’s the often missed intervention in quality trauma care.’

Panelists’ used scientific evidence to select care criteria relevant to patient outcomes such as specific time thresholds for treatment. The source of evidence for the majority of discussions was unclear (76%), with a portion derived from the systematic review performed in preparation for the workshop (12%) or panelists own research, studies conducted at their respective centers, or other unpublished work (12%). Panelists’ comments approximately equally supported (53%) and disputed (47%) the evidence discussed based on their own research (13%), knowledge of others research including published literature (40%), critiques of study methodology or the amount of evidence available (32%), and clinical experience (14%). In cases of weak or absent scientific evidence their expertise provided face validity for certain treatments, protocols and time thresholds:

‘The timing I’m not sure about, there’s no class one evidence to support it…there are cohort studies…but there are so many confounders in this research…. At the same time…there is some value added to early management…prevention of certain secondary complications, skin management, managing the neurogenic bowel and bladder, interaction with the family…that’s why I advocate for it….’

Panelists’ with data and quality of care measurement backgrounds used their expertise to bring attention to potential challenges with data collection and measurement and proposed methods to circumvent possible problems. For example, panelists suggested developing new data systems by linking existing databases that have previously not communicated with one another:

‘…if you linked your blood dispatch with your trauma registry you could actually [record] activation [time] of your massive [blood] transfusion protocol…it’s something you could measure.’

Patient perspectives were also considered (although the panel did not include patients). This was often done in conjunction with consideration of clinical outcomes and primarily in relation to time thresholds for treatment.

Speaker one: ‘[Surgical fixation of] fractures can and often [are left for] 48 hours which is poor care. It is not well received by patients or families…it may lead to complications like DVT [deep vein thrombosis], etc.… So I think 48 hours for operative interventions for fractures is a useful indicator.’

Speaker two: ‘What’s the evidence-base for early treatment of non-compound fractures?’

Speaker three: ‘It exists for the femur and it exists for the pelvis. Apart from that…for more peripheral fractures there’s really none…. The question is whether you limit [the indicator] to those [fractures] with an evidence base.’

Speaker two: ‘But, if we’re interested in patient oriented outcomes…and it is definitive operative intervention…[then] I still think…that the 48 hours, from the patient’s perspective it would be a reasonable quality indicator.’

Most of the two-day discussion involved an integration of both clinical perspectives on the impact proposed indicators may have on patient outcome(s), and methodological perspectives on key properties of quality measures (indicator reliability, validity, and measurability). Indicators ranked highly by the panel were often those that satisfied both perspectives.

### Contextual considerations and variation

There was substantial discussion on variations in health system organization and patient populations that exist between centers. Panelists highlighted these variations and discussed their impact on the relevance and applicability of indicators to different centers. Modifications to language, data elements, and other components were considered as ways to increase generalizability of the indicators. If an indicator was not broadly relevant, it was not considered sufficiently important to be selected for development.

Differences in provider training, service provision, and organization by systems were sources of variation that were discussed. Some differences were perceived to influence the value of a proposed indicator (e.g., organization of operating rooms), while others were not (e.g., availability of local practice guidelines).

Other sources of variability were the differing populations and communities to which hospitals provide their services. These differences were discussed in two ways. First, as a barrier to the comparability of indicator results, consequently limiting the possibility of creating system wide benchmarks.

‘…this is going to reflect where these people live though. Nothing to do with the trauma system. A community is full of MRSA [Methicillin-resistant *Staphylococcus aureus*] so the hospital that looks after these people will have a huge rate [of MRSA infections] versus one that’s in a less affected part of the country.’

Second, as a factor affecting the relevance of certain indicators across trauma centers:

‘…penetrating urban trauma is such a small proportion of total trauma for most citizens outside of maybe five major US cities…I don’t know whether it’s worth having a separate indicator for it.’

Despite a lack of comparability, the panelists agreed that significant clinical problems warranted implementing quality indicators that could be used for quality improvement within individual hospitals. These would be in keeping with a standardized set of quality indicators that were broadly applicable, but would be used to track a center’s own performance over time:

‘…It’s very important to have infection control. It’s hard to compare rates across countries and even within a city, but, it’s more a process you follow for good infection control practice…C diff [*Clostridium difficile*]…reflects not only community prevalence but a lot of hospital issues…. It’s an important cause for a quality indicator.’

Standardization of language and data elements was a major focus of discussion. This was in part due to different classification systems for illness severity (e.g., how is major injury or persistent hemorrhagic shock operationally defined). Panelists highlighted the importance of standardization to ensure the capture of reliable and comparable data by end users.

The ability to discern reasons for outliers was considered during discussions of benchmarking. One method identified was to exclude patients for whom an indicator would not be entirely applicable, thereby improving indicator specificity. A second proposed method was to design indicators to be sensitive and flag suspect cases for further review to determine the reasons for why quality indicator criteria were not met.

### Data collection and management

The collection, management, and review of data were frequently discussed. Panelists often considered the end-users’ ability to collect and interpret data and thus these issues were crucial when considering the implementation of quality indicators. For example, the inadequacy of data collection systems and registries was emphasized as a challenge in implementing certain indicators*.*

In particular, concerns were raised about timely access to data (‘…The data we get from the…coroner is generally three years out of date…timeliness may be an issue in all of this discussion and you may want to bear that in mind.’) and the lack of integration between existing databases held by different stakeholders (e.g., no current linkage between most coroners databases and trauma registries). The absence of important, relevant data in existing databases was also identified as a barrier: ‘…The national trauma registry doesn’t contain a lot of these indicators [or] the data to support these indicators.’

These barriers stimulated discussion of potential new sources of data (‘the other rich source…is the workers compensation board. They’ve got a really rich database on injury-related death’), opportunities to integrate existing data systems to access new information (‘…all you need to do is form a link with the medical examiner and [they’ve] got the data…. ’), and collaborations that may improve the timeliness of data access (‘…our trauma registry personnel actually worked in the medical examiners office and so we actually produce an annual up to date death data report…. ’).

The quality of medical record documentation and of the data abstracted from these records was identified as an important deficit in healthcare that threatens the reliability and validity of indicator results. Support for quality measures targeting the quality of data was provided by healthcare measurement and data management experts: ‘…Anything that encourages the whole trauma system to…improve the data that is submitted…to have a measure that forces the issue that we need better data collection…. ’

With regards to making indicators operational and practical to implement, the panel members agreed that national acceptance of the indicators was necessary, but that data collection and management should be at the levels of states and provinces: ‘…Have it down at a [state or] provincial level which is perhaps more meaningful given that everything is within the context of [state and] provincial administration.’

Panelists from all countries (Canada, US, Australia) agreed this may be the best approach. Another aspect of data collection considered for nearly every indicator was the ease for end users to capture appropriate information. Panelists’ tried to precisely define all data elements, including the patient populations and diseases/treatments, to enable easy implementation and reliable data collection, and to improve the sensitivity and specificity of indicators. Many of the panel members commented that explicit details were necessary for valid and comparable measurements of quality of care and a lack of detail was a factor in their low ratings of select indicators such as the ‘time to treatment of spine injury’:

‘I don’t think it’s a good indicator, [the appropriate time to treatment] depends [on] what you’re talking about; spinal cord injury, spinal column injury, [or both] spinal cord [and] spinal column injury. Some [patients]…need…[surgery] or immobilization, some actually don’t. So, [the indicator is] very broad [as are] the scales to judge if something needs to be treated.’

## Discussion

We used a constant comparative method of analysis to analyze the transcripts of a consensus panel workshop convened to create a standardized set of quality indicators for injury care. Four main themes were identified in discussion of the selection, development, and implementation of standardized quality indicators: specifying a clear purpose and goal(s) for the indicators to ensure relevant data were included, and that indicators could be used for system-wide benchmarking and improving patient outcomes; incorporating evidence, expertise, and patient perspectives to identify important problems that may benefit from quality measurement and address potential measurement challenges; considering context and variations between centers in the health system that could influence the relevance or application of an indicator (*i.e.*, case mix adjustment) such as service organization, provider training, and patient populations; and contemplating data collection and management issues, including availability of existing data sources (e.g., types of data and linkage potential), quality of data (including data sources such as medical record documentation), timeliness of data abstraction, and the potential role for primary data collection.

Frameworks and protocols for developing and evaluating quality indicators have been proposed [7,11,12,18]. Published guides have been primarily informed through literature reviews, expert opinion, and the retrospective examination of the successes and failures of indicator implementation [7,9,11,12,18,19]. To our knowledge, this was the first analysis of key considerations that emerge from the selection and development of quality indicators by an expert panel using a modified RAND/UCLA Appropriateness method. Our findings complement the existing literature on developing and implementing quality indicators in healthcare. First, framing discussions of quality indicator development is essential. Our panel framed their deliberations with the goal of developing quality indicators to improve patient outcomes (not the structure or processes of care). This goal reflects a primarily clinical perspective on quality. Stakeholder perspectives will influence quality measurement. Previous studies have demonstrated that the composition of consensus panels does influence ratings. Differences in judgment have been shown based on physician specialty [20], between mixed and single-specialty physician panels [21,22], and between mixed physician and non-physician panels [23]. We elected to have a multi-disciplinary panel comprised of clinical experts, quality of care experts, trauma program managers, and trauma registry stakeholders. The panel adopted a predominately clinical view on quality (clinical outcome) with some consideration given to patient experience and cost-effectiveness. Other possible outcomes of quality measurement include increasing accountability of healthcare providers and systems and informing consumer choice. Explicitly outlining quality-reporting goals at the onset of an indicator development process is likely to facilitate indicator development. Furthermore, stakeholders must specify whether an indicator’s intended use will be for system-wide benchmarking or internal quality improvement. This distinction shaped discussions in our study around variations between centers that could influence implementation of indicators and comparability of reported results. The AHRQ asks panelists to evaluate the usefulness of an indicator in terms of internal quality improvement and center comparability [7]. Finally, those developing quality indicators may consider pairing structural and process indicators. This coincides with a key component of the RAND/UCLA Appropriateness method; linking the treatment/indication under discussion to patient benefit [24].

Second, using expert opinion to supplement scientific evidence has been previously used successfully to develop quality indicators [25,26]. This has been primarily done to identify relevant problems in care that would benefit from quality measurements. For example, Crandall *et al.* reported a lack of consensus on quality-of-care indicators for Irritable Bowel Syndrome after implementing the indicators across multiple sites [27]. The indicators were perceived as irrelevant in many centers given differences in treatment preferences and patient populations. Thus, a multi-disciplinary, multi-institutional, and multi-national panel may be a great asset. Gaining the insight of varying perspectives on the magnitude of problems at differing centers/systems enables indicators to be selected and developed accordingly. Furthermore, a diversity of expertise may also be important when there are evidence gaps in the literature. For example, our analysis showed that 12% of discussions regarding evidence were of research not included in the systematic review informing the workshop, and included panelists’ own research, studies conducted at their respective centers, or other unpublished work. Diversity of expertise was essential for our panel’s development of quality indicators using ‘evidence-informed expert opinion.’ Incorporating patient perspectives into quality indicator development is also increasingly recognized as important [28]. In our study, patients and families were not included on the panel, although patients’ perspectives were discussed by the experts. However, Gagliardi *et al.* have demonstrated that experts may not adequately represent the needs and preferences of patients when it comes to selecting performance measures [29]. Therefore, alternative strategies to incorporate patient perspectives may need to be considered. For example, Bokhour *et al.* used focus groups to generate patient-centered indicators and presented them to clinical experts for evaluation [28].

Third, our core theme of ‘variation and contextual considerations’ has been highlighted by other researchers. Specifically, there is a tension between developing simple, broadly applicable quality indicators and measures that can be responsive to individual contexts and capture clinically relevant variation. To complicate matters, inconsistent terminology in medical charting [7], classification systems, and databases within and between countries [5] have been identified as important considerations in ensuring comparability of indicator results. In developing their quality indicators, the AHRQ requires panelists to consider the extent to which indicators were subject to bias in terms of systematic differences that would affect the indicator in a way not related to quality of care [7]. Higashi *et al.* recommend a few alternatives to this approach including narrowly specifying the population of interest to limit the number of possible exceptions and mandating documentation for why indicated care was not provided [30]. Our panel proposed restricting quality indicator development to those indicators that could only be broadly implemented. However, panelists recognized that not even all ‘broadly’ applicable indicators will be relevant to all stakeholders and consistently measured. Hence, they proposed identifying a smaller group of core indicators essential for quality measurement within a broader indicator menu from which stakeholders could select.

Fourth, concerns regarding data collection and management identified in this study are common in the literature. A study by Klazinga *et al.* on performance indicator research and benchmarking in Europe emphasized the lack of database linkage, quality of documentation, and lack of information in registries as key barriers to advancing performance indicator research [5]. Furthermore, other researchers have encountered data collection challenges when implementing new sets of quality indicators. These include, but are not limited to poor documentation [30], necessary data not collected in current clinical practice [27], and ambiguous definitions of data elements resulting in unreliable data collection [27]. Recommendations have been made to resolve some of these issues. For example, the American College of Cardiologists and American Heart Association (ACC/AHA) published a methodological guide for quality indicator development in which they advise to clearly and precisely define data elements, including target populations and avoid long lists of inclusion/exclusion criteria, to make implementation easier [18]. The ACC/AHA also suggests evaluating indicators based on data availability and quality, whereby those indicators that can use available high-quality data are of greater value [18]. Other quality indicator development programs, such as the AHRQ, require panelists to evaluate the likelihood that the data necessary for an indicator is available in medical charts [7]. However, our panel proposed an alternative approach, focusing on developing the ‘best’ indicators possible regardless of data availability. Panel members reasoned that quality indicator development can also be used to motivate important changes in data collection.

Fifth, while the key themes identified in our study are broadly consistent with published frameworks for quality indicator development, they highlight a lack of consensus between different indicator development protocols. For example, Campbell *et al.*[11] proposed a protocol for indicator development and testing where clarity of definitions, necessity, and validity were the primary focus of panel evaluation and selection, while implementation issues (acceptability, unintended consequences), reliability, and feasibility were the focus of pilot-testing. Conversely, the AHRQ has suggested that experts consider data availability during initial expert indicator evaluation (data collection and management) and the consistency of clinical terminology (variation and contextual considerations) [7]. These variations in evaluation frameworks highlight both the need to prospectively consider the key considerations for quality indicator development identified in our study prior to starting indicator development and the potential value of creating standardized criteria for quality indicator development and evaluation [7,11].

The results of our study need to be considered within the context of its limitations. First, our study was an analysis of the deliberation of a single panel and the generalizability of the themes is unknown. Although, the themes identified in our study are consistent with the literature, empirical evaluation is needed to determine if providing this guidance to quality indicator developers in advance of panel discussion can help produce valid and reliable quality indicators in an efficient manner. Second, the expert panel was tasked with developing indicators to measure the quality of care for critically injured adult patients in high-income countries. The experiences of others who have developed quality indicators for other medical conditions, patient populations, or healthcare systems may differ. However, the overarching challenges and considerations of indicator development presented in this paper are likely common across healthcare. Third, the expert panel was comprised of multidisciplinary stakeholders in injury care, but did not include patients or family members. Although panel participants considered patient perspectives during quality indicator development their views may differ from those of patients and families. This highlights the potential trade-off of facilitating consensus by minimizing panel diversity against ensuring representation by all relevant stakeholders.

## Conclusions

Development of evidence-informed quality indicators is a first step towards measuring and improving the quality of healthcare. Our study identified four broad considerations to guide development of quality indicators: specifying a clear purpose and goals for the quality indicators; incorporating evidence, expertise, and patient perspectives; considering context and variation; and contemplating data collection and management. Our study provides a description of the key themes of discussion among a panel of clinical, managerial, and data experts developing quality indicators. Consideration of these themes could help shape deliberation of future panels convened to develop quality indicators.

## Competing interests

Sharon Straus is an Associate Editor for Implementation Science and all editorial decisions regarding this manuscript were made by another editor. None of the authors have financial or professional conflicts of interest that would influence the conduct or reporting of this study. Mr. Bobrovitz, Ms. Parrilla and Dr. Stelfox had full access to all of the data in the study and take responsibility for the integrity of the data and the accuracy of the data analysis.

## Authors’ contributions

NB, JSP, SES, and HTS contributed to the study concept and design. JSP, NB, and HTS acquired the consensus panel recordings. NB, JSP, HTS, MS, and SES analyzed and interpreted the data. NB and JSP drafted the initial manuscript. JSP, NB, HTS, MJS, and SES made critical revisions to the manuscript and approved the final draft. HTS provided administrative, technical, and material support. HTS acted as the study supervision. All authors read and approved the final manuscript.

## Supplementary Material

Additional file 1 Coding framework. Click here for file

Additional file 2 Description of the coding framework. Click here for file

## References

[B1] Institute of Medicine To Err Is Human: Building a Safer Health System 2000 Washington, D.C: National Academy Press

[B2] Committee on the Quality of Health Care in America Institute of Medicine Crossing the Quality Chasm: A new Health System for the 21st Century 2001 Washington DC: National Academy Press

[B3] Baker GR Norton PG Flintoft V Blais R Brown A Cox J Etchells E Ghali WA Hébert P Majumdar SR The Canadian adverse events study: the incidence of adverse events among hospital patients in Canada CMAJ 2004 170 11 1678 1686 15159366 10.1503/cmaj.1040498 PMC408508

[B4] Groene O Skau JK Frolich A An international review of projects on hospital performance assessment Int J Qual Health Care 2008 20 3 162 171 10.1093/intqhc/mzn008 18339665

[B5] Klazinga N Fischer C ten Asbroek A Health services research related to performance indicators and benchmarking in Europe J Health Serv Res Policy 2011 16 2 38 46 10.1258/jhsrp.2011.011042 21737528

[B6] Nadzam DM Turpin R Hanold LS White RE Data-driven performance improvement in health care: the joint commission’s Indicator Measurement System (IMSystem) Jt Comm J Qual Improv 1993 19 11 492 500 8313012 10.1016/s1070-3241(16)30030-x

[B7] Farquhar M Hughes R AHRQ Quality Indicators Patient Safety and Quality: An Evidence-Based Handbook for Nurses 2008 Rockville, MD: Agency for Healthcare Research and Quality 341 367 21328752

[B8] Stelfox HT Bobranska-Artiuch B Nathens A Straus SE Quality indicators for evaluating trauma care: a scoping review Arch Surg 2010 145 3 286 295 10.1001/archsurg.2009.289 20231631

[B9] Lorenz KA Lynn J Dy S Wilkinson A Mularski RA Shugarman LR Hughes R Asch SM Rolon C Rastegar A Quality measures for symptoms and advance care planning in cancer: a systematic review J Clin Oncol 2006 24 30 4933 4938 10.1200/JCO.2006.06.8650 17050878

[B10] Stelfox HT Straus SE Nathens A Bobranska-Artiuch B Evidence for quality indicators to evaluate adult trauma care: a systematic review Crit Care Med 2011 4 846 859 21317653 10.1097/CCM.0b013e31820a859a

[B11] Campbell SM Kontopantelis E Hannon K Burke M Barber A Lester HE Framework and indicator testing protocol for developing and piloting quality indicators for the UK quality and outcomes framework BMC Fam Pract 2011 12 85 10.1186/1471-2296-12-85 PMC3176158 21831317

[B12] Kötter T Blozik E Scherer M Methods for the guideline-based development of quality indicators--a systematic review Implement Sci 2012 7 21 10.1186/1748-5908-7-21 PMC3368783 22436067

[B13] Santana MJ Stelfox HT Straus S Development and evaluation of the evidence-informed quality indicators for adult injury care Arch Surg 2012 10.1097/SLA.0b013e31828df98e 23657078

[B14] Santana MJ Stelfox HT Quality indicators used by trauma centres for performance measurement J Trauma 2012 72 5 1298 1302 10.1097/TA.0b013e318246584c 22673258

[B15] Zhang Y Wildemuth BM Wildemuth B Qualitative analysis of content Applications of Social Research Methods to Questions in Information and Library Science edn 2009 Santa Barbara, CA: Greenwood Press 308 319

[B16] Clark JP Godlee F, Jefferson T How to peer review a qualitative manuscript Peer Review in Health Sciences 2003 2 London: BMJ Books 219 235

[B17] Donabedian A Evaluating the quality of medical care Milbank Q 2005 83 691 729 10.1111/j.1468-0009.2005.00397.x 16279964 PMC2690293

[B18] Spertus JA Eagle KA Krumholz HM American College of Cardiology and American Heart Association methodology for the selection and creation of performance measures for quantifying the quality of cardiovascular care J Am Coll Cardiol 2005 45 1147 1156 10.1016/j.jacc.2005.03.011 15808779

[B19] Seow H Snyder CF Shugarman LR Mularski RA Kutner JS Lorenz KA Wu AW Dy SM Developing quality indicators for cancer end-of-life care: proceedings from a national symposium Cancer 2009 115 17 3280 3289 10.1002/cncr.24439 19514090

[B20] Kahan JP Park RE Leape LL Bernstein SJ Hilborne LH Parker L Kamberg CJ Ballard DJ Brook RH Variations by specialty in physician ratings of the appropriateness and necessity of indications for procedures Med Care 1996 34 5 512 523 8656718 10.1097/00005650-199606000-00002

[B21] Coulter I Adams A Shekelle P Impact of varying panel membership on ratings of appropriateness in consensus panels: a comparison of a multi- and single disciplinary pane Health Ser Res 1995 30 577 591 PMC1070076 7591782

[B22] Leape LL Park RE Kahan JP Brook RH Group judgments of appropriateness: the effect of panel composition Qual Assur Health Care 1992 4 151 159 1511149

[B23] Campbell SM Hann M Roland MO Quayle J Shekelle PG The effect of panel membership and feedback on ratings in a two-round Delphi survey: results of a randomized controlled trial Med Care 1999 37 964 968 10.1097/00005650-199909000-00012 10493474

[B24] Fitch K Bernstein SJ Aguilar MD Burnand B LaCalle JR Lá zaro P van het Loo M McDonnell J Vader JP Kahan JP The RAND/UCLA Appropriateness Method User’s Manual 2001 Santa Monica, CA: RAND Corporation

[B25] Shekelle PG MacLean CH Morton SC Wenger NS ACOVE quality indicators Ann Intern Med 2001 135 8 653 667 11601948 10.7326/0003-4819-135-8_part_2-200110161-00004

[B26] Wenger NS Shekelle PG Assessing care of vulnerable elders: ACOVE project overview Ann Intern Med 2001 135 2 642 646 11601946 10.7326/0003-4819-135-8_part_2-200110161-00002

[B27] Crandall WV Boyle BM Colletti RB Margolis PA Kappelman MD Development of process and outcome measures for improvement: lessons learned in a quality improvement collaborative for pediatric inflammatory bowel disease Inflamm Bowel Dis 2011 17 10 2184 2191 10.1002/ibd.21702 21456033

[B28] Bokhour BG Pugh MJ Rao JK Avetisyan R Berlowitz DR Kazis LE Improving methods for measuring quality of care: a patient-centered approach in chronic disease Med Care Res Rev 2009 66 2 147 166 10.1177/1077558708327174 19074307

[B29] Gagliardi A Lemieux-Charles L Brown A Sullivan T Goel V Stakeholder preferences for cancer care performance indicators Int J Health Care Qual Assur 2008 21 2 175 189 10.1108/09526860810859030 18578202

[B30] Higashi T Lessons learned in the development of process quality indicators for cancer care in Japan Biopsychosoc Med 2010 4 14 10.1186/1751-0759-4-14 PMC2990721 21054836

