# Supplementary material for: A qualitative analysis of a consensus process to develop quality indicators of injury care
Source: Implement Sci. 2013 Apr 18;8:45. doi: 10.1186/1748-5908-8-45 (PMC3639212; doi:10.1186/1748-5908-8-45)
Supplement: Additional file 1 — Coding framework. [file 1748-5908-8-45-S1.doc]

APPENDIX 1. CODING FRAMEWORK

Table 1. Coding framework including progression from codes and categories to themes.

| **Themes** | **Categories** | **Codes** |
| --- | --- | --- |
| Specifying a clear purpose and goal(s) for the indicators to ensure relevant data used for system-wide benchmarking and improving patient outcomes. | 1. **Purpose**: identifying the purpose of each quality indictor and of a standardized set of quality indicators | 1. Accountability, aspirational indicators, clarifying conceptual focus, definitive vs. attempted treatment, discerning high vs low quality, filling knowledge gaps, generalizable, high-income vs. low-income countries, informing reform, local vs. system-wide improvement, mapping care vs. time thresholds, primary, secondary prevention, parsimony vs. capturing specifics, quality improvement practices, surrogate measures, responsive vs. preventative measurement, unintended consequences, prompting action/review, patient vs provider vs system level |
|  | 1. **Prioritizing**: identifying the value of the indicator in the context or local, global priorities | 2. Appropriateness, disciplinary bias, local vs regional priorities, feasibility, magnitude of the problem/necessity, measurability vs. conceptual focus, patient-centered view, prioritizing data elements |
|  | 1. **Ideal healthcare**: identifying what an ideal standard is and which indicators/indicator elements could flag those cases of ideal versus sub-standard care | 3. Achievable standards, aspirational care, benchmarking and standardized practice, coordinated care, critical thinking and decision making, effective, communication and collaboration, reporting diligence, documented care, enforcement of regulation and self-policing, frequent of assessment, integration, matching resources to patient need, organization, patient outcome(s), timely care, optimal performance, public/lay people considerations, thresholds (time and other) |
|  | 1. **Ideal system-wide quality improvement and assurance**: identifying important elements to affect change at the system level | 4. Comparability, consideration of existing guidelines, core indicators, cost/feasibility, knowledge translation, criteria matching/deviating from accreditation standards, reporting methods |
| Incorporating evidence, expertise and patient perspectives to identify important clinical problems and potential measurement challenges. | 1. **Evidence**: evidence brought up in the discussion | 1. Review literature, non-review literature, indicators based on well-established protocols, age of evidence, link to patient outcome, feasibility of obtaining evidence, evidence gaps and opportunities for inquiry, time and other thresholds |
|  | 1. **Supporting or refuting**: panelists supporting or refuting the evidence discussed | 2. Supporting, refuting |
|  | 1. **Expertise**: panelist experience with the indicators or with the evidence presented, and rationale for supporting or refuting the evidence discussed | 3. Speakers own research, knowledge of local research or the literature, critique of methodology or the amount of evidence, anecdotal based on speakers academic or clinical experiences, operationalizing indicators, indicator adherence, professional society support, understanding and comprehension |
|  | 1. **Perspective**: identifying indicator stakeholders and discussing their perspectives of the indicators’ scope and aim | 4. Patient-centered care, multi-disciplinary perspective, perspective of different phases of care |
| Considering context and variations between centres in the health system that could influence either the relevance or application of an indicator. | 1. **Developments and evolutions in healthcare**: considering the history, current state and progression of practice and technology in relevant domains of care and their impact on the indicators | 1. Current context, background/origin of the indicator, current vs. historical practice, out-of-date, clinical dogma, reform, relevance in current practice, technological advances and capabilities |
|  | 1. **Variability in centers and systems**: identifying how variations in settings, populations, care practice and training, and resources will influence indicator value and implementation | 2. Patient group priorities, administration and management, center volume, burden distribution, confounders and bias, defining procedures, physical geographic variability, local procedure guidelines and protocols, low-income vs. high-income countries, political landscapes, varying priorities and agendas, systematic trends in service provision, reporting and documentation, variation in clinical training, variations in information systems, variation in patient classification |
| Contemplating data collection and management issues including availability of existing data sources, quality of data, timeliness of data abstraction and the potential role for primary data collection. | 1. **Components of the indicator:** key components in “building” the indicator | 1. Collapsing/amalgamating, defining procedures, patient eligibility, flexibility, inclusion/exclusion criteria, presentation format, defining level of application, measurability, periodicity of measurement, parsimony vs. capturing specifics, structure/process, time threshold |
|  | 1. **Indicator data collection and analysis:** defining the necessary components involved in indicator data collection and analysis | 2. Access/feasibility, data elements, defining data elements, devising a data analysis method, devising a data collection method, timeliness of data abstraction, integrating data systems, quality of the data collected, need for primary data collection, reasons for outliers vs. poor quality, prospective versus retrospective collection, sentinel events, subjectivity in measurement |
|  | 1. **Language:** key language considerations and their consequences on the indicator | 3. Antiquated or dated language, capturing conceptual focus, communication and collaboration, connotation, procedural definitions, interpretation by end users |
|  | 1. **Sensitivity/specificity:** variables that would impact the sensitivity and specificity of the indicator | 4. Classification of patient groups, numerator and denominator eligibility, omission, population confounding factors, subjectivity in measurement |
|  | 1. **Indicator application**: how indicator operates once applied and implemented | 5. Discrepancies, overlap with accreditation and guidelines, practicalities in care provision |
|  | 1. **Support:** support for the implementation process and maintaining operation of the quality indicators | 6. Local support for quality measurement and improvement, political and financial support for measurement, policy mandates, professional society recommendations, provincial drive, public/lay support, human resources |
