# Supplementary material for: A qualitative analysis of a consensus process to develop quality indicators of injury care
Source: Implement Sci. 2013 Apr 18;8:45. doi: 10.1186/1748-5908-8-45 (PMC3639212; doi:10.1186/1748-5908-8-45)
Supplement: Additional file 2 — Description of the coding framework. [file 1748-5908-8-45-S2.doc]

APPENDIX 2. DESCRIPTION OF THE CODING FRAMWORK

**Theme 1:** Specifying a clear purpose and goal(s) for the indicators to ensure relevant data elements were included, and that indicators could be used for system-wide benchmarking and improving patient outcomes.

**Categories:**

1. **Purpose**: identifying the purpose of each quality indictor and of a standardized set of quality indicators.
2. **Prioritizing**: identifying the value of the indicator in the context or local, global priorities.
3. **Ideal healthcare**: identifying what an ideal standard is and which indicators/indicator elements could flag those cases of ideal versus sub-standard care.
4. **Ideal system-wide quality improvement and assurance**: identifying important elements to affect change at the system level.

**Categories and Associated Codes:**

1. **Purpose**: identifying the purpose of each quality indictor and of a standardized set of quality indicators.

- Accountability: making providers and administrators accountable for the care they provide
- Aspirational indicators: ideal quality indicators that could be broadly applied to bring about system-wide change
- Clarifying conceptual focus: (includes teasing apart related issues) of each individual indicator
- Definitive vs. attempted treatment: defining the aim of the indicator to address definitive care or attempted treatment, which can still indicate organization
- Discerning high vs low quality: (formerly comparability through standardization) ability to distinguish between centers with good quality of care and poor quality of care
- Filling knowledge gaps: (includes triggering further investigation, includes highlighting knowledge gaps, includes mapping care) indicators designed to map existing care, identifying areas for new research or filling epidemiological knowledge gaps in the literature
- Generalizable: (formerly generalizability)
- High-income vs. low-income countries: target for indicator use
- Informing reform: to drive the improvement of care
- Local vs. system-wide improvement: (includes local vs wide-spread benchmarking, includes application level) determining whether the purpose is local or systematic improvement
- Mapping care vs. time thresholds: is the purpose to fill knowledge gaps or benchmark quality using time thresholds
- Primary, secondary prevention: (formerly prevention) indicator focus
- Parsimony vs. capturing specifics: (includes core indicators, includes parsimony) identifying key aspects of care addressed by a small core set of indicators as opposed to measuring many components of patient outcome with many indicators
- Quality improvement practices: indicators designed to inform quality improvement practices
- Surrogate measure: indicator acting as a surrogate measure for a latent characteristic or quality
- Responsive vs. preventative measurement: indicator measured to respond to known sub-standard care or map care with uncertain variation
- (un)intended consequences (prompting appropriate action or review vs. forcing inappropriate action)
- Prompting action/review: (including prompting review): to change care practices
- Patient vs provider vs system level: (formerly system benefit vs. individual patient benefit, formerly system-wide measure)

1. **Prioritization**: identifying the factors and motivations influencing what aspects of care are selected for measurement and how they are measured. Also, assessing the value of the indicator with respect to varying priorities.

- Appropriateness (of indicator based on improvement needs)
- Disciplinary bias: (formerly bias) biases about what should be prioritized and measured based on disciplinary favoritism
- Local vs regional priorities
- Feasibility: prioritizing feasibility of measurement over what could ideally be captured
- Magnitude of the problem/necessity
- Measurability vs. conceptual focus: determining whether an element of care addressed by an indicator is important enough to warrant resolving issues with its measurability
- Patient-centered view: patient-centered care as a health care priority
- Prioritizing data elements: to ensure parsimony within indicator definitions and target key patient groups

1. **Ideal healthcare**: identifying ideal standards of care and which indicators/indicator elements could flag those cases of ideal versus sub-standard care.

- Achievable standards: discussing whether the proposed standards of care are achievable
- Aspirational care: high standards that may not be feasible given resource constraints etc…
- Benchmarking and standardized practice: (includes guidelines) requirement of high quality care
- Coordinated care: (formerly care principles)
- Critical thinking and decision making: (formerly care principles) on the part of healthcare providers and administrators
- Effective: (formerly care principles)
- Communication and collaboration: between providers and components of the healthcare system
- Reporting diligence: (formerly diligence, includes lack of reporting, includes negligence/omission)
- Documented care: (formerly documentation) care is well documented
- Enforcement of regulation and self-policing
- Frequent of assessment: (formerly frequency/regularity of assessment) frequent assessment is ideal
- Integration: incorporating indicators into common practice
- Matching resources to patient need
- Organization: in the system, sharing information, etc.
- Patient outcome(s): improving patient outcomes
- Timely care: (formerly timeliness)
- Optimal performance
- Public/lay people considerations: including the public in identifying healthcare goals
- Thresholds (time and other): determining ideal standards with impact on patient outcome

1. **Ideal system-wide quality improvement and assurance**: identifying important components to affect change at the system level.

- Comparability: of quality measurement data necessary for improvement at a system level
- Consideration of existing guidelines: (formerly considering existing guidelines) integrating existing guidelines into indicator development
- Core indicators (common group all hospitals would use)
- Cost/feasibility: feasibility of implementation costs so multiple centers may implement quality indicators
- Knowledge translation: (includes clear dissemination) disseminating conference results and indicator results once implemented, incorporating stakeholders from the beginning
- Criteria matching/deviating from accreditation standards
- Reporting methods: common method of reporting required

**Theme 2:** Incorporating evidence, expertise and patient perspectives to identify important clinical problems and potential measurement challenges.

**Categories:**

1. **Evidence**: evidence brought up in the discussion.
2. **Supporting or refuting**: panelists supporting or refuting the evidence discussed.
3. **Expertise**: panelist experience with the indicators or with the evidence presented, and rationale for supporting or refuting the evidence discussed.
4. **Perspective**: identifying indicator stakeholders and discussing their perspectives of the indicators’ scope and aim.

**Categories and Associated Codes:**

1. **Evidence**: used to aid/supplement the selection/development of indicators.

- Review literature: scientific evidence from the literature review preceding the conference
- Non-review literature: scientific evidence not from the literature review
- Indicators based on well-established protocols: (includes parsimonious indicator to follow well-established guidelines)
- Age of evidence
- Link to patient outcome: (includes causality, patient outcome, usefulness of measure): necessary identification of a causal link between a specific service and patient outcome
- Feasibility of obtaining evidence: to support an indicator if there is a current lack of evidence
- Evidence gaps and opportunities for inquiry: (including knowledge gaps, Diligence, investigation, opportunities for research inquiry) gaps in existing evidence that represent opportunities for research inquiry to support a particular link between outcome and a process or structural indicator
- Time and other thresholds: (includes usefulness of thresholds) identification of evidence to justify specific time thresholds and other thresholds

1. **Supporting or refuting**: panelists’ discussion supporting or refuting the evidence discussed.

- Supporting: (includes significance – of evidence)
- Refuting: (includes significance – of evidence)

1. **Expertise**: panelist experience with the indicators or with the evidence presented, and rationale for supporting or refuting the evidence discussed.

- Speakers own research
- Knowledge of local research or the literature: (includes evidence contradicting old practice)
- Critique of methodology or the amount of evidence: (includes strength, validity, face-validity vs lack of evidence) discussions of the validity and strength of evidence supporting or refuting an indicator
- Anecdotal based on speakers academic or clinical experiences: (includes clinical argument vs. evidence, includes lack of evidence vs. clinical judgment, face-validity, likelihood, variations in clinical judgment, expectancy/expectation)
- Operationalizing indicators: (includes efficiency, effectiveness, efficacy, includes experience using the indicator) panelists speaking about how operational an indicator would be in practice, based on their experience using the indicator
- Indicator adherence: (formerly probability of indicator implementation and adherence, includes probability) based on panelist experience using an indicator
- Professional society support: whether specialized clinical societies would support an indicator based on the alignment with current guidelines and practices
- Understanding and comprehension: panel members’ level of familiarity with an indicator

1. **Perspectives**: impact of different stakeholder perspectives in developing an indicator.

- Patient-centered care: (includes care matched to patient needs) valuable in indicator development; panelists putting themselves into patients’ shoes
- (Multi)-disciplinary perspective: highlighting key problems with measurement and compliance with indicators based on disciplinary knowledge and experience
- Perspective of different phases of care: pre-hospital, in-hospital post-hospital

**Theme 3:** Considering context and variations between centres in the health system that could influence either the relevance or application of an indicator.

**Categories:**

1. **Developments and evolutions in healthcare**: considering the history, current state and progression of practice and technology in relevant domains of care and their impact on the indicators.
2. **Variability in centers and systems**: identifying how variations in settings, populations, care practice and training, and resource will influence indicator value and implementation.

**Categories and Associated Codes:**

1. **Developments and evolutions in healthcare**: considering the history, current state and progression of practice and technology in relevant domains of care and their impact on the indicators.

- Current context: ability to implement the indicator in current care systems
- Background/origin of the indicator
- Current vs. historical practice
- Out-of-date: (formerly dated) indicators and practices
- Clinical dogma: adherence to old practices
- Reform: forcing progress of care
- Relevance in current practice: relevance of an indicator in current healthcare settings
- Technological advances and capabilities: influence these changes will have on indicator data elements including diagnostic tests, common procedures, and time thresholds used.

1. **Variability in centers and systems**: identifying how variations in settings, populations, care practice and training, and resource will influence indicator value and implementation.

- Patient group priorities: (includes inclusion vs exclusion, includes care priorities for different patient groups, includes patient population priorities): identifying unique needs of specific patient populations and tailoring the indicator to address those needs
- Administration and management: variability in structure of healthcare systems
- Center volume
- Burden distribution: different burden of problems affect different geographical areas
- Confounders and bias: (includes environmental confounding factors) in sampling groups due to environmental variability
- Defining procedures: definitions of procedures varies between centers and systems
- Physical geographic variability: (formerly geography variability) affecting pre-hospital indicators and transfer indicators
- Local procedure guidelines and protocols: (formerly (local) considerations, includes protocol variation)
- Low-income vs. high-income countries
- Political landscapes: influencing structure of health care delivery
- Varying priorities and agendas: (formerly priorities and agendas)
- Systematic trends in service provision: what is commonly done is some systems compared to others
- Reporting and documentation: (includes public vs. private reporting)
- Variation in clinical training: (formerly training variation)
- Variations in information systems: data collection – registries
- Variation in patient classification: (formerly variation in methods of specification for population): major trauma, injury severity score, poly-trauma

**Theme 4:** Contemplating data collection and management issues including availability of existing data sources, quality of data, timeliness of data abstraction and the potential role for primary data collection.

**Categories:**

1. **Components of the indicator:** key components in “building” the indicator.
2. **Indicator data collection and analysis:** defining the necessary components involved in indicator data collection and analysis.
3. **Language:** key language considerations and their consequences on the indicators.
4. **Sensitivity/specificity:** variables that would impact the sensitivity and specificity of the indicator.
5. **Indicator application**: how indicator operates once applied and implemented.
6. **Support:** support for the implementation process and maintaining operation of the quality indicators**.**

**Categories and Associated Codes:**

1. **Components of the indicator**: key components in “building” the indicator.

- Collapsing/amalgamating: (formerly convergence/amalgamation) of multiple/redundant indicators
- Defining procedures
- Patient eligibility: (formerly eligibility, inclusive data elements, inclusion vs exclusion) inclusion criteria
- Flexibility: building flexibility into the indicator to account for small variability between centers and thus be integrated at the local level, yet have generalizable, comparable results
- Inclusion/exclusion criteria
- Presentation format: (includes format) presentation format for the indicator results
- Defining level of application: (formerly indicator level) patient, hospital or system level
- Measurability: built to make sure indicator is measurable
- Periodicity of measurement: define time intervals for assessment
- Parsimony vs. capturing specifics: details of data elements
- Structure/process: structure indicators should be tied to process indicators because patient outcome is influenced by the adherence to protocols not the existence of protocols
- Time threshold: the required timeframe for providing a specific service

1. **Indicator** **Data Collection** **and Analysis**: defining the necessary components involved in indicator data collection and analysis.

- Access/feasibility: (includes convenience, effort intensiveness of data collection, feasibility) to data collection and analysis
- Data elements: e.g. list to give a sense of indicator purpose
- Defining data elements: to ensure reproducibility
- Devising a data analysis method
- Devising a data collection method: if data not available or currently collected
- Timeliness of data abstraction
- Integrating data systems: local vs. provincial vs. national data databases/registries
- Quality of the data collected: (includes accuracy) validity and reliability
- Need for primary data collection
- Reasons for outliers vs. poor quality: being able to explain outliers must be a part of the indicator otherwise a label of poor quality may be given inappropriately
- Prospective versus retrospective collection: (formerly retrospective)
- Sentinel events
- Subjectivity in measurement

1. **Language**: key language considerations and their consequences on the indicators.

- Antiquated, dated language: not relevant to current practice
- Capturing conceptual focus: (formerly capture conceptual focus) so the indicator is understood and reproducible
- Communication and collaboration: key for service providers to use the same language
- Connotation: consequence of language, has to be explicit and simple so that people are all doing the same thing and not interpreting data elements differently
- Procedural definitions: (formerly defining procedures) precise use of language to ensure minimal interpretation errors
- Interpretation by end users: simple language so people can understand

1. **Sensitivity/specificity**: variables that would impact the sensitivity and specificity of the indicator.

- Classification of patient groups: (includes usefulness of patient classification thresholds) classification systems e.g. injury severity scores scores
- Numerator and denominator eligibility: (formerly denominator, numerator, includes eligibility, inclusion/exclusion criteria, specifying the population)
- Omission: omitting false positives
- Population confounding factors
- Subjectivity in measurement: subjective measurement more prone to issues with sensitivity and specificity

1. **Indicator application**: how indicator operates once applied and implemented.

- Discrepancies: discrepancy between the indicators intended purpose and how it is actually used, regulation of its use
- Overlap with accreditation and guidelines: integrated in practice
- Practicalities in care provision: the practicalities you’ll have to consider for the indicator to function appropriately in practice)

1. **Support**: support for the implementation process and maintaining operation of the quality indicators.

- Local support for quality measurement and improvement: (includes compliance/receptiveness, includes administration and management)
- Political and financial support for measurement
- Policy mandates: mandating indicator adoption, periodic use
- Professional society recommendations: (formerly provider organization recommendations of care) recommending that the indicators be used
- Provincial drive: to be the governing body in charge of implementing indicators
- Public/lay support: (formerly public/lay people considerations) achieving public support
- Human resources: (includes accessible) required for implementation and sustainable use
